# Inhibition of CISD2 enhances sensitivity to doxorubicin in diffuse large B-cell lymphoma by regulating ferroptosis and ferritinophagy

**DOI:** 10.3389/fphar.2024.1482354

**Published:** 2024-11-13

**Authors:** Chaofeng Zhang, Siting Zhan, Yanjun He, Zhiqun Pan, Zhongyi You, Xiongpeng Zhu, Qi Lin

**Affiliations:** ^1^ Department of Hematology and Rheumatology, the Affiliated Hospital of Putian University, Putian, Fujian Province, China; ^2^ Department of Haematology, Quanzhou First Hospital Affiliated to Fujian Medical University, Quanzhou, China; ^3^ School of Basic Medical Science, Putian University, Putian, Fujian Province, China; ^4^ Department of Pharmacy, The Affiliated Hospital of Putian University, Putian, Fujian Province, China; ^5^ Key Laboratory of Translational Tumor Medicine in Fujian Province, Putian University, Putian, Fujian Province, China

**Keywords:** diffuse large B-cell lymphoma, CDGSH iron sulfur domain 2, ferroptosis, ferritinophagy, drug resistance, Doxorubicin

## Abstract

**Background:**

CDGSH iron-sulfur domain 2 (CISD2), an iron-sulfur protein with a [2Fe-2S] cluster, plays a pivotal role in the progression of various cancers, including Diffuse Large B-cell Lymphoma (DLBCL). However, the mechanisms by which CISD2 regulates the occurrence and development of DLBCL remain to be fully elucidated.

**Methods:**

The potential role of CISD2 as a predictive marker in DLBCL patients treated with the R-CHOP regimen was investigated through bioinformatics analysis and clinical cohort studies. DLBCL cell lines (SUDHL-4 and HBL-1) were employed in this research. Adenoviral (AV) plasmids were used to either silence or overexpress CISD2 in these DLBCL cell lines. Additionally, the induction of ferroptosis in DLBCL cell lines was assessed. Various parameters, including cell proliferation, intracellular free iron levels, lipid peroxides, reactive oxygen species (ROS), and mitochondrial membrane potential (MMP), were measured. Furthermore, the expression of proteins associated with ferroptosis and ferritinophagy was analyzed. Drug-resistant DLBCL cell lines were developed by gradually increasing doxorubicin (DOX) concentration over 6 months. The biological role of CISD2 in these drug-resistant DLBCL cell lines was subsequently assessed.

**Results:**

Elevated CISD2 levels were found to be associated with decreased sensitivity of DLBCL patients to the R-CHOP regimen, as indicated by bioinformatics and clinical cohort analysis. Silencing CISD2 significantly reduced cell proliferation, increased iron accumulation, depleted glutathione (GSH), and elevated malondialdehyde (MDA) levels, alongside the accumulation of ROS and increased MMP. Additionally, BECN1 and NCOA4 expressions were upregulated, while p62, FTH1, and GPX4 expressions were downregulated. Conversely, overexpression of CISD2 reversed these effects. Treatment of DLBCL cell lines with Erastin led to decreased CISD2 levels. Notably, in drug-resistant DLBCL cell lines, CISD2 knockdown promoted ferroptosis and ferritinophagy, restoring sensitivity to DOX and enhancing the efficacy of Erastin treatment.

**Conclusion:**

Our findings suggest that CISD2 may play a role in the drug resistance observed in DLBCL patients. Inhibition of CISD2 could enhance ferroptosis and ferritinophagy, potentially improving the sensitivity of DLBCL cells to DOX treatment.

## Introduction

Annually, approximately 150,000 new cases of diffuse large B-cell lymphoma (DLBCL) are diagnosed globally each year, representing about one-third of all newly diagnosed non-Hodgkin lymphoma (NHL) cases (Susanibar-Adaniya and Barta, 2021). The introduction of the R-CHOP immunochemotherapy regimen—comprising rituximab, cyclophosphamide, vincristine, doxorubicin (DOX), and prednisone—has significantly enhanced patient outcomes and marks a key milestone in targeted therapy for DLBCL (Sarkozy and Sehn, 2018; Wang, 2023). However, around 40% of patients experience treatment failure, either due to resistance or relapse, and many of these individuals fail to respond to second-line therapies, often leading to a poor prognosis. In recent years, extensive research has addressed the heterogeneity of DLBCL, prompting the development of novel strategies for more accurate pathological subtyping and treatment of this lymphoma (Miao et al., 2019; Takahara et al., 2023). Targeted small molecule drugs, including BTK inhibitors such as ibrutinib and zanubrutinib, the BCL2 inhibitor venetoclax, and PD-1 monoclonal antibodies, have shown efficacy in certain DLBCL subtypes. Despite these advancements, a substantial number of DLBCL patients still fail to benefit from these treatments (Nowakowski et al., 2019; Cherng and Westin, 2021; Wilson et al., 2021). As a result, ongoing exploration of novel molecular targets and signaling pathways in DLBCL remains critical for the advancement of precision treatment in this lymphoma.

Generally, CDGSH iron-sulfur domain 2 (CISD2), also known as NAF-1, ZCD2, or Miner1, is predominantly localized to the outer mitochondrial membrane but is also present in the endoplasmic reticulum (ER) membrane (Chen et al., 2009; Shen et al., 2021). This dual localization enables CISD2 to interact with multiple organelles and coordinate key biological processes, including cell proliferation, apoptosis, autophagy, and ferroptosis in various tumors (Liao et al., 2021; Zhang et al., 2023a). Previously, several studies have shown that the upregulation of CISD2 is linked to the development and progression of various aggressive malignancies and is associated with poor clinical outcomes across multiple cancer types (Kim et al., 2018; Liao et al., 2021; Zhang et al., 2023a). Conversely, downregulation or knockdown of CISD2 expression has been shown to inhibit tumor growth and improve the efficacy of cancer treatments (Wang et al., 2014; Kim et al., 2018). Our previous studies indicate that elevated CISD2 expression serves as both a reliable diagnostic factor and an unfavorable prognostic marker in DLBCL(Zhang et al., 2023b). However, the precise regulatory role of CISD2 in DLBCL remains to be further elucidated.

Collectly, this study aims to investigate the role of CISD2 in the onset and progression of DLBCL. The expression and function of CISD2 in DLBCL through bioinformatics and clinical cohort analysis were performed, and the role of CISD2 in regulating ferroptosis and ferritinophagy in DLBCL were explored through inhibition and overexpression of CISD2 *in vitro*. Additionally, we examined whether CISD2 influences drugy resistance to DLBCL patients treatment. Taken together, our results provided evidence that CISD2 is involved in the regulation of ferroptosis and ferritinophagy in DLBCL and contributes to treatment resistance.

## Materials and methods

### Bioinformatics analysis

The raw expression profiles and clinical data from GSE117556 (n = 928) were downloaded from the Gene Expression Omnibus (GEO, https://www.ncbi.nlm.nih.gov/geo/) (Sha et al., 2019). The correlation between differential CISD2 expression levels in DLBCL patients and their clinical features was analyzed.

### Patients collection

This study was reviewed and approved by the Ethics Committee of Quanzhou First Hospital Affiliated to Fujian Medical University (ID: [2023]K096). We retrospectively collected data from DLBCL patients who were hospitalized with complete records at our institution from January 2017 to January 2024. The collected medical records included age, gender, treatment regimen, prognosis, International Prognostic Index (IPI), Ann Arbor stage, and paraffin-embedded tissue sections. Inclusion criteria were: ① Pathological diagnosis based on the 2016 revision of the WHO classification of lymphoid neoplasms (Swerdlow et al., 2016); ② Complete clinical records, including treatment details. Exclusion criteria were: ① Patients with other tumors, immunodeficiency diseases, or infectious diseases; ② Patients with incomplete clinical records.

### Cell culture and treatment

The DLBCL cell lines SUDHL-4 (obtained from Meisen, China) and HBL-1 (a gift from Fujian Medical University Union Hospital) were selected and cultured in RPMI-1640 medium (Hyclone, USA) supplemented with 10% fetal bovine serum (FBS, Gibco, USA), 1% streptomycin, and penicillin (Hyclone, United States) in an incubator (ThermoFisher, United States) with 5% CO₂ at 37°C. Adenovirus (AV) plasmid, a linear double-stranded DNA virus without an envelope, can infect both dividing and non-dividing cells by entering through receptor-mediated endocytosis without integrating into the host cell genome. In this study, the AV plasmids were provided by Zolgene, China, with the sequence for silencing CISD2 (shCISD2) detailed in Supplementary Table S1. The AV sequence for CISD2 overexpression (oeCISD2) was as follows: ATG​GTG​CTG​GAG​AGC​GTG​GCC​CGT​ATC​GTG​AAG​GTG​CAG​CTC​CCT​GCA​TAT​CTG​AAG​CGG​CTC​CCA​GTC​CCT​GAA​AGC​ATT​ACC​GGG​TTC​GCT​AGG​CTC​ACA​GTT​TCA​GAA​TGG​CTT​CGG​TTA​TTG​CCT​TTC​CTT​GGT​GTA​CTC​GCA​CTT​CTT​GGC​TAC​CTT​GCA​GTT​CGT​CCA​TTC​CTC​CCG​AAG​AAG​AAA​CAA​CAG​AAG​GAT​AGC​TTG​ATT​AAT​CTT​AAA​ATA​CAA​AAG​GAA​AAT​CCG​AAA​GTA​GTG​AAT​GAA​ATA​AAC​ATT​GAA​GAT​TTG​TGT​CTT​ACT​AAA​GCA​GCT​TAT​TGT​AGG​TGT​TGG​CGT​TCT​AAA​ACG​TTT​CCT​GCC​TGC​GAT​GGT​TCA​CAT​AAT​AAA​CAC​AAT​GAA​TTG​ACA​GGA​GAT​AAT​GTG​GGT​CCA​CTA​ATA​CTG​AAG​AAG​AAA​GAA​GTA. Additionally, empty vectors (shCON or oeCON) were used as negative controls. The DLBCL cell lines were transfected with the constructed AV plasmids for 6 h. After 24 h of transfection, the cells were used for further studies.

### Construction of drug-resistant cell lines

The HBL-1 cells were subsequently used to induce drug-resistant cell lines through a stepwise increase in doxorubicin (DOX, MedChemExpress, USA) concentration. DOX was initially administered at a concentration of 0.4 nM, with daily monitoring. The same concentration of DOX was applied 6–8 times repeatedly until the HBL-1 cells stabilized at this level. Gradually, the DOX concentration was increased to 8 nM, and after approximately 6 months, drug-resistant cell lines (HBL-1/DOX) were successfully established. The HBL-1/DOX cells were then treated with shCISD2 and analyzed in further studies.

### Quantitative real-time polymerase chain reaction (qRT-PCR)

Total RNA from the cells was isolated using TRIzol reagent (Invitrogen, United States), and then transcribed into cDNA using the HiScript Q RT SuperMix for qPCR (Vazyme, China). qRT-PCR was performed on the CFX Connect Real-Time PCR Detection System (BioRad, United States) using HQ SYBR qPCR Mix (without ROX) (Zomanbio, China). CISD2 levels were quantified using the 2^−△△Ct^ method, with GAPDH as a loading control. The primer sequences are provided in Supplementary Table S2.

### Cell proliferation examination

The DLBCL cell lines were transfected with AV plasmids and adjusted to a cell suspension (1 × 10^6^ cells/mL). A total of 180 µL of the cell suspension was plated into each well of a 96-well plate, including blank and control wells, with three replicates per condition. Next, 20 µL of CCK8 reagent (Zomanbio, China) was added to each well and incubated for 4 h. The optical density (OD) was measured using a microplate reader (ThermoFisher, USA) at wavelengths of 450 nm (detection) and 620 nm (reference). The cell proliferation was calculated using the following formula: Cell proliferation (%) = [OD (test) - OD (blank)]/[OD (Control) - OD (blank)] × 100%. The half-maximal inhibitory concentration (IC50) was determined to assess the response of CISD2 knockdown/overexpression DLBCL cells to DOX and to evaluate the inhibitory efficacy of DOX on HBL-1 and HBL-1/DOX cells.

### Iron assay

First, approximately 1 × 10^6^ cells were harvested and lysed. The supernatants were then collected after centrifugation at 4°C. Following the instructions of the iron assay kit (Solarbio, China), standard solutions were prepared and mixed with the collected supernatants. Chloroform (Sinopharm, China) was then added to the mixtures, which were vortexed for 5 min. After a second round of centrifugation, the upper phase was collected, and the OD was measured at 593 nm using a spectrophotometer (Meipuda, China).

### Antioxidant system detection

In this study, total glutathione (GSH, Solarbio, China) and malondialdehyde (MDA, Solarbio, China) levels in treated cells were detected using a colorimetric method. After extraction with a pre-prepared solution, the cells were disrupted by sonication on ice. The supernatants were collected after centrifugation and kept on ice for further analysis. According to the kit instructions, the OD of each sample was measured at 532 nm and 600 nm using a spectrophotometer (Meipuda, China), and the concentrations of GSH and MDA in the cells were calculated.

### Flow cytometry

Reactive oxygen species (ROS) and mitochondrial membrane potential (MMP) in treated cells were detected using flow cytometry (BD Biosciences, United States). For ROS detection, approximately 5 × 10^5^ cells in 1 mL RPMI-1640 medium (Hyclone, United States) were mixed with 1 µL of DCFH-DA solution (Solarbio, China) and incubated for 40 min. The cells were then centrifuged, collected, and ROS levels were measured immediately. For MMP detection, a single-cell suspension of 0.5 mL (approximately 5 × 10^5^ cells) was prepared, and 0.5 mL of JC-1 staining working solution (Solarbio, China) was added. The mixture was incubated for 20 min, followed by two washes with JC-1 buffer solution. The cells were then resuspended in 1 mL of JC-1 buffer solution and measured immediately.

### Western Blotting (WB) assay

The treated cells were collected and lysed using RIPA lysis buffer (Beyotime, China) supplemented with 1% PMSF (Solarbio, China). Total protein was extracted, and its concentration was measured using a BCA protein assay kit (Beyotime, China). The levels of the following proteins were then detected: CISD2 (Cat. 66082-1-PBS, Proteintech, China), BECN1 (Cat. K101553P, Solarbio, China), p62 (Cat. RM4519, Biodragon, China), FTH1 (Cat. YT1692, Immunoway, USA), NCOA4 (Cat. YT0302, Immunoway, United States), and GPX4 (Cat. BD-PN3047, Biodragon, China). β-actin (Cat. YT0099, Immunoway, USA) was used as a loading control.

### Statistical analysis

Experimental data analysis was performed using R programming (version 4.2), and plots were generated using the ggplot2 package. Measurement data conforming to a normal distribution and homogeneity of variance were expressed as mean ± SEM and compared using the Student’s t-test or one-way analysis of variance (ANOVA). A *P*-value of <0.05 was considered statistically significant.

## Result

### Elevated CISD2 levels were associated with resistance to the R-CHOP regimen in DLBCL

**FIGURE 1:**
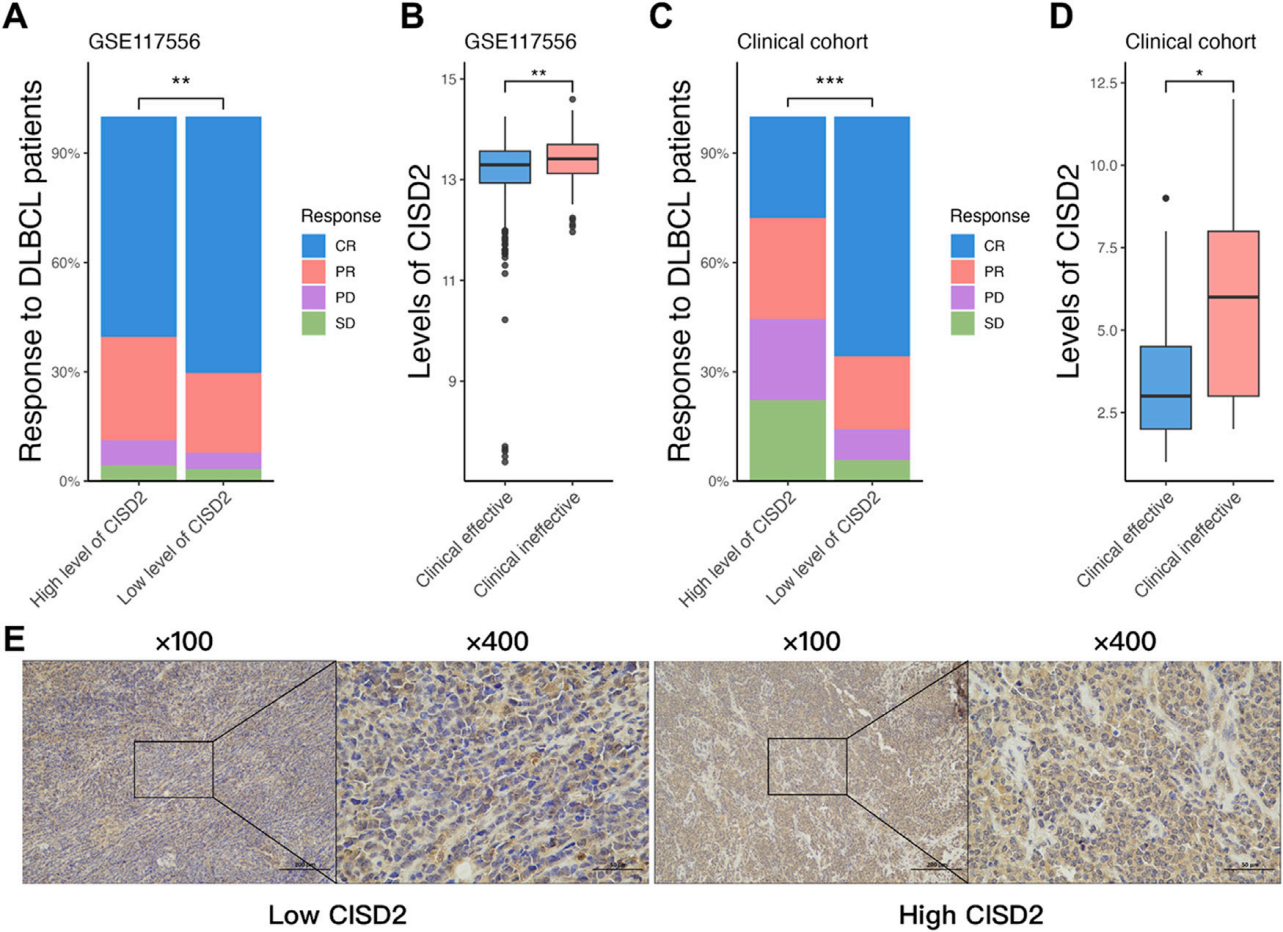
Elevated CISD2 levels were associated with resistance to the R-CHOP regimen in DLBCL. **(A)** The comparison of the response of R-CHOP treated DLBCL patients with high and low level of CISD2 in GSE117556 dataset. **(B)** The comparison of the level of CISD2 between clinical effectiveness and clinical ineffectiveness in GSE117556 dataset. **(C)** The comparison of the response of R-CHOP treated DLBCL patients with high and low level of CISD2 in our clinical cohort. **(B)** The comparison of the level of CISD2 between clinical effectiveness and clinical ineffectiveness in our clinical cohort. **(E)** CISD2 expression in DLBCL Clinical Cohort using IHC Staining. DLBCL, Diffuse large B-cell lymphoma; IHC, Immunohistochemical. Results were expressed as mean ± SE (n = 3). *P < 0.05, **P < 0.01, ***P < 0.001.

A total of 844 patients were extracted from the GSE117556 dataset (Sha et al., 2019), all of whom were primarily treated with the R-CHOP regimen after excluding those with insufficient clinical data. Among these patients, 60.43% of those with high CISD2 levels achieved complete remission (CR), compared to 70.38% of patients with low CISD2 levels, as determined by the Wilcoxon Rank Sum Test (Figure 1A, *P* < 0.05). As shown in Figure 1B (*P* < 0.05), DLBCL patients who achieved clinical efficacy (including CR and partial remission) exhibited lower CISD2 levels than those with clinical inefficacy (including progressive disease and stable disease). Additionally, 53 DLBCL patients from our clinical cohort were included, with their clinical characteristics presented in Table 1. Immunohistochemical (IHC) assays were performed on formalin-fixed paraffin-embedded (FFPE) tissue sections stained with a CISD2-specific monoclonal antibody (Cat. 66082-1-PBS, Proteintech, China). IHC staining was assessed, with scores below 6 categorized as low CISD2 levels and higher scores as high CISD2 levels, as shown in Figure 1E. Furthermore, DLBCL patients with high CISD2 levels in our cohort (Figures 1C, D, *P* < 0.05) demonstrated a similar trend in clinical response to the R-CHOP regimen as observed in the GSE117556 dataset. These findings indicate that high CISD2 levels are associated with a worse clinical response to the R-CHOP regimen.

**TABLE 1:** Basic information of the DLBCL clinical cohort included in the study.

Clinical characteristics	High level of CISD2	Low level of CISD2	*P*-value
Age (<60 years/≥60 years)	6/12	16/19	0.5673
Gender (Male/Female)	8/10	15/20	1
COO subtype (GCB/non-GCB)	6/12	23/12	0.0510
IPI(<2/≥2)	4/14	14/21	0.0204

### CISD2 was involved in the regulation of ferroptosis and ferritinophagy in DLBCL

Previous studies (Li et al., 2022; Zhang et al., 2023a; Shangguan et al., 2024) suggest that CISD2 may play a role in promoting tumor cell proliferation and drug resistance through ferroptosis and ferritinophagy (Li et al., 2022). In this study, we investigated the regulatory role of CISD2 in the occurrence and development of DLBCL. DLBCL cell lines (SUDHL-4 and HBL-1) were transfected with constructed AV plasmids (AV plasmid structure shown in Supplementary Figure S1A). To identify the optimal shRNA targeting CISD2, three different shRNAs (shRNA1, shRNA2, and shRNA3) were transfected into the DLBCL cell lines, with shCON serving as the control. qRT-PCR results demonstrated that shRNA2 significantly inhibited the relative expression of CISD2 (Supplementary Figure S1B, *P* < 0.05). Consequently, shRNA2 (shCISD2) was selected for subsequent experiments. Additionally, the relative expression of CISD2 in DLBCL cell lines was increased through the transfection of oeCISD2 (Supplementary Figure S1C, *P* < 0.05). The transfection efficiency of shCISD2, shCON, oeCISD2, and oeCON was observed under a fluorescence microscope (Nikon, Japan). As shown in Supplementary Figure S1D, the AV plasmids effectively transfected the DLBCL cell lines. The protein expression of CISD2 was significantly influenced by AV plasmid transfection (Supplementary Figure S1D, *P* < 0.05).

**FIGURE 2:**
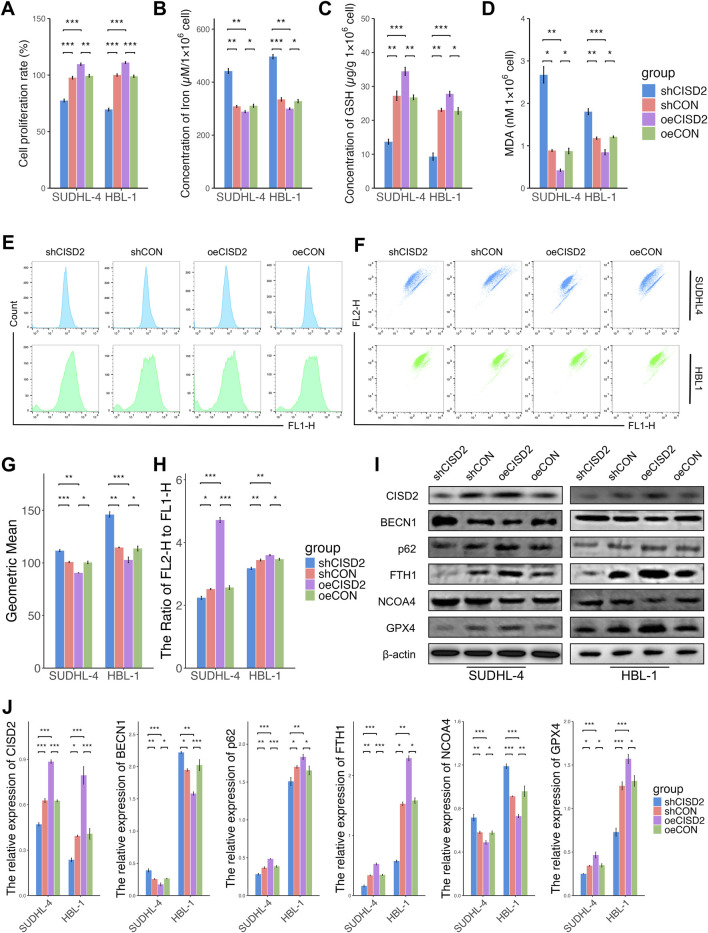
CISD2 was involved in the regulation of ferroptosis and ferritinophagy in DLBCL. **(A)** The cell proliferation rate of DLBCL cell lines after knockdown/overexpression of CISD2 (left. SUDHL-4; right, HBL-1). **(B)** The iron levels were evaluated (left. SUDHL-4; right, HBL-1). **(C)** The GSH levels were detected (left. SUDHL-4; right, HBL-1). **(D)** The MDA levels were analyzed (left. SUDHL-4; right, HBL-1). **(E, G)** Intracellular ROS levels in DLBCL cell lines were detected though flow cytometry. **(F, H)** The MMP levels in DLBCL cell lines were detected though flow cytometry. **(I)** Schematic diagram of WB results. **(J)** Statistical analysis of relative protein. DLBCL, Diffuse large B-cell lymphoma; GSH, glutathione; MDA, malondialdehyde; MMP, mitochondrial membrane potential; ROS, Reactive oxygen species; WB, Western Blotting. Results were expressed as mean ± SE (n = 3). *P < 0.05, **P < 0.01, ***P < 0.001.

The role of CISD2 in regulating ferroptosis and ferritinophagy in DLBCL was investigated. Knockdown of CISD2 resulted in decreased cell proliferation of DLBCL cell lines compared with shCON (Figure 2A, *P* < 0.05). Conversely, CISD2 overexpression significantly increased cell proliferation compared to cells transfected with oeCON or shCISD2 (Figure 2A, *P* < 0.05). Silencing CISD2 led to an increased concentration of cellular iron in DLBCL cell lines (Figure 2B, *P* < 0.05), whereas overexpression of CISD2 reduced iron concentration (Figure 2B, *P* < 0.05). Antioxidant systems were also affected by changes in CISD2 expression. As shown in Figures 2C, D, GSH levels decreased in DLBCL cell lines transfected with shCISD2, while MDA levels increased (*P* < 0.05). In contrast, oeCISD2 enhanced GSH levels and reduced MDA levels compared with the oeCON or the shCISD2 group (*P* < 0.05). Similarly, shCISD2 induced ROS generation in DLBCL cell lines compared to the shCON (Figures 2E, G *P* < 0.05), while ROS production was inhibited by oeCISD2 (Figures 2E, G, *P* < 0.05). Decreased mitochondrial membrane potentials (MMPs) were observed in DLBCL cell lines with CISD2 knockdown, whereas MMPs increased with CISD2 overexpression (Figures 2F, H, *P* < 0.05). Additionally, the expression of CISD2, and proteins related to ferroptosis and ferritinophagy was assessed (Figures 2I, J). Transfection of shCISD2 inhibited the expression of CISD2, p62, FTH1, and GPX4 (*P* < 0.05) while enhancing BECN1 and NCOA4 expression (*P* < 0.05) compared with shCON group. On the other hand, oeCISD2 transfection resulted the opposite effects compared with oeCON gourp (*P* < 0.05). These findings indicate that modulation of CISD2 affects ferroptosis and ferritinophagy in DLBCL.

### Induction of ferroptosis was associated with inhibition of CISD2 in DLBCL

**FIGURE 3:**
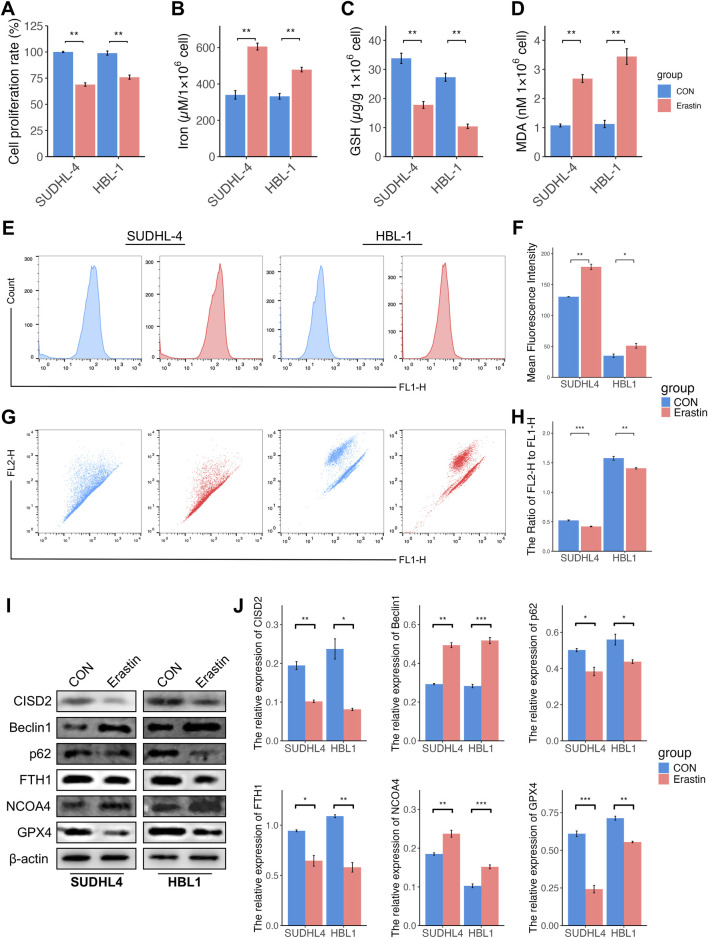
Induction of ferroptosis was associated with inhibition of CISD2 in DLBCL. **(A)** The cell proliferation rate of DLBCL cell lines treated by Erastin (left. SUDHL-4; right, HBL-1). **(B)** The iron levels were evaluated (left. SUDHL-4; right, HBL-1). **(C)** The GSH levels were detected (left. SUDHL-4; right, HBL-1). **(D)** The MDA levels were analyzed (left. SUDHL-4; right, HBL-1). **(E, F)** Intracellular ROS levels in DLBCL cell lines were detected though flow cytometry. **(G, H)** The MMP levels in DLBCL cell lines were detected though flow cytometry. **(I)** Schematic diagram of WB results. **(J)** Statistical analysis of relative protein. DLBCL, Diffuse large B-cell lymphoma; GSH, glutathione; MDA, malondialdehyde; MMP, mitochondrial membrane potential; ROS, Reactive oxygen species; WB, Western Blotting. Results were expressed as mean ± SE (n = 3). *P < 0.05, **P < 0.01, ***P < 0.001.

To explore the relationship between ferroptosis and CISD2 levels, Erastin, a known ferroptosis inducer that mediates ferroptosis through various pathways (Sato et al., 2018), was used to access. Previous studies (Chu et al., 2019; Wu et al., 2019) have demonstrated that 10 µM Erastin effectively induces ferroptosis in DLBCL cell lines. Treatment with Erastin resulted in a significant decrease in cell proliferation (Figure 3A, *P* < 0.05), an increase in iron concentration (Figure 3B, *P* < 0.05), a reduction in GSH levels (Figure 3C, *P* < 0.05), and an elevation in MDA levels (Figure 3D, *P* < 0.05). Erastin also stimulated ROS generation (Figures 3E, F, *P* < 0.05) and decreased MMPs (Figures 3G, H, *P* < 0.05). Interestingly, exposure to Erastin inhibited CISD2 levels (Figures 3I, J, *P* < 0.05). Additionally, the protein levels of p62, FTH1, and GPX4 were downregulated, while the levels of BECN1 and NCOA4 were upregulated (Figures 3I, J, *P* < 0.05). These findings indicate that Erastin treatment induces ferroptosis and ferritinophagy, accompanied by the inhibition of CISD2.

### Knockdown of CISD2 increased the sensitivity of HBL1/DOX cells to DOX

**FIGURE 4:**
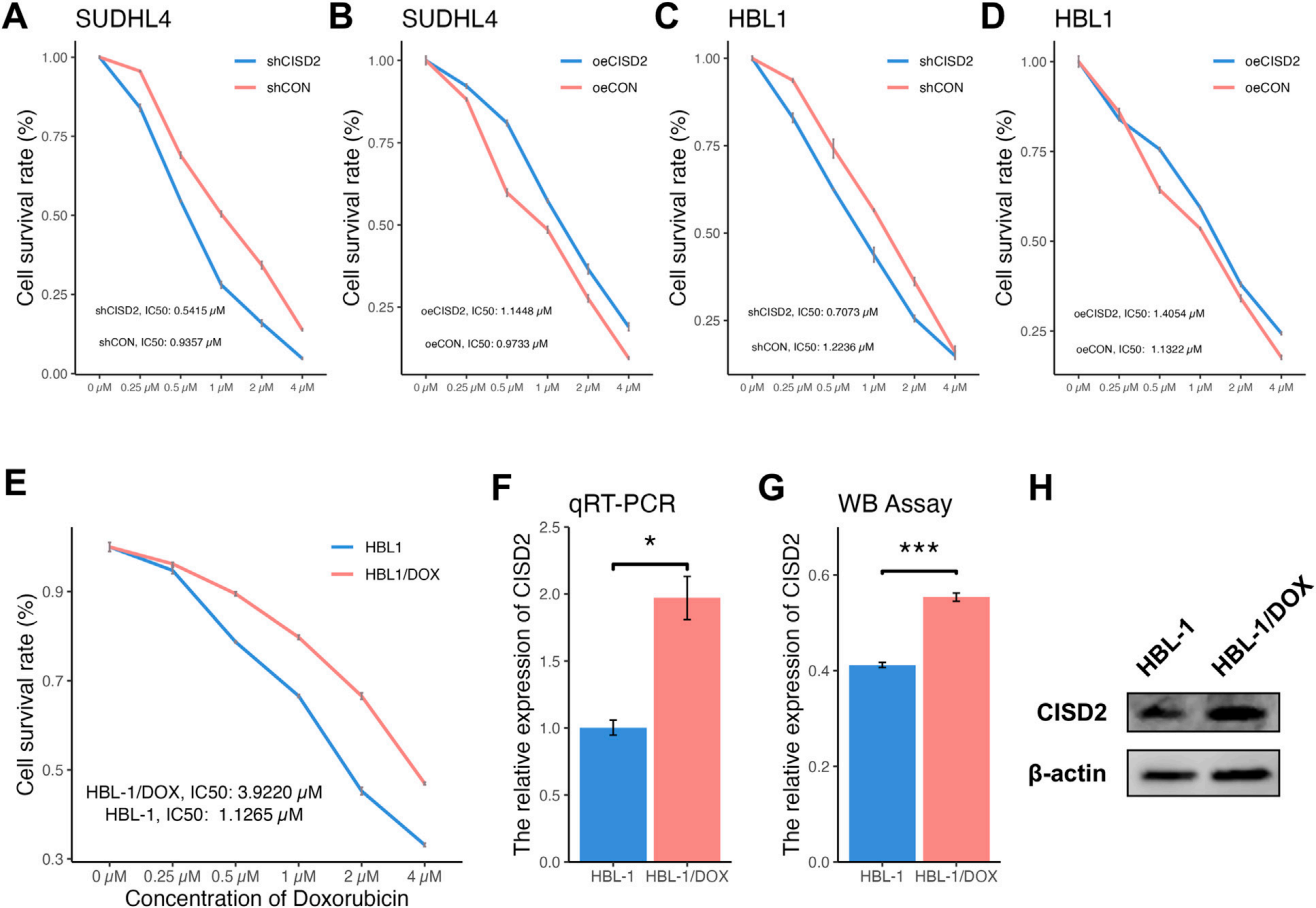
Differentially concentration of DOX treated DLBCL cell lines and the drug resistance of DLBCL cell line was built. The CCK8 method was used to detect the changing trend of cell proliferation rate with DOX concentration after inhibition/overexpression of CISD2 **(A, B)** SUDHL-4 cell lines, **(C, D)** HBL-1 cell lines). **(E)** The cell proliferation rate after shCISD2 treatment of HBL-1/DOX cell lines change trend with differential DOX concentration. **(F)** qRT-PCR detection was used to detect the relative expression of CISD2 in HBL-1 and HBL-1/DOX. **(G)** WB method was used to detect the relative expression of CISD2 in HBL-1 and HBL-1/DOX. **(H)** Schematic diagram of WB results. DLBCL, Diffuse large B-cell lymphoma; DOX, Doxorubicin; qRT-PCR, quantitative real-time polymerase chain reaction; WB, Western Blotting. Results were expressed as mean ± SE (n = 3). *p < 0.05, **P < 0.01, ***P < 0.001.

To assess the impact of CISD2 on DOX sensitivity, DLBCL cell lines were transfected with shCISD2, oeCISD2, or empty vectors (snCISD2 or onCISD2) and exposed to varying concentrations of DOX. As shown in Figures 4A, C, treatment with shCISD2 led to a significant decrease in the IC50 values (SUDHL-4: 0.5415 µM, HBL-1: 0.7073 µM) compared to cells treated with snCISD2 (SUDHL-4: 0.9357 µM; HBL-1: 1.2236 µM). Conversely, overexpression of CISD2 increased the resistance of DLBCL cell lines to DOX (SUDHL-4: 1.1448 µM; HBL-1: 1.4054 µM, Figures 4B, D) compared with transfection of onCISD2 (SUDHL-4: 0.9733 µM; HBL-1: 1.1322 µM, Figures 4B, D). These results suggest that modulation of CISD2 levels affects the sensitivity of DLBCL cell lines to DOX.

HBL-1/DOX cells, which were developed through incremental exposure to DOX over 6 months, exhibited more pronounced clustering compared to HBL-1 cells (Supplementary Figure S2A). Sensitivity tests revealed that HBL-1/DOX cells had a higher IC50 (3.9220 µM) compared to HBL-1 cells (1.1265 µM) when exposed to different DOX concentrations (Figure 4E). CISD2 expression levels were higher in HBL-1/DOX cells compared to HBL-1 cells, as confirmed by both qRT-PCR (Figure 4F, *P* < 0.05) and WB assay (Figures 4G, H, *P* < 0.05), indicating that CISD2 plays a critical role in DOX resistance.

**FIGURE 5:**
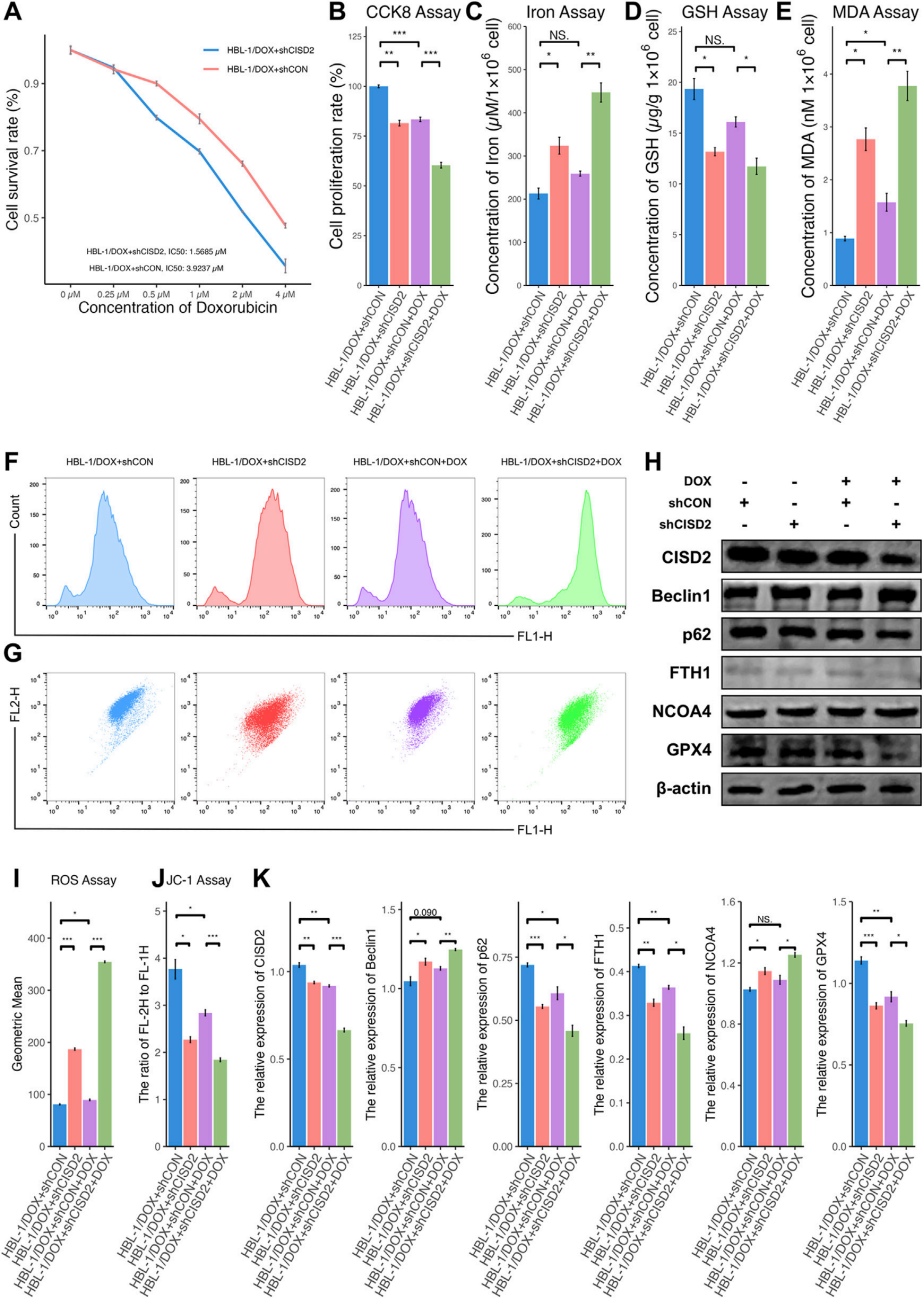
Knockdown of CISD2 increased the sensitivity of HBL-1/DOX cells to DOX. **(A)** CCK8 method was used to detect the changing trend of cell proliferation rate with DOX concentration after shCISD2/shCON treats HBL-1/DOX cells; **(B)** The cell proliferation rate after shCISD2 treated HBL-1/DOX cells changes. **(C)** Spectrophotometry was used to detect intracellular iron ion levels. **(D)** GSH levels were detected. **(E)** MDA levels were detected. **(F, I)** Intracellular ROS levels in HBL-1/DOX cells treated by shCISD2 were detected though flow cytometry. **(G, J)** The MMP levels in HBL-1/DOX cells treated by shCISD2 were detected though flow cytometry. **(H)** WB method was used to detect the relative expression of CISD2 in HBL-1 and HBL-1/DOX. **(K)** Schematic diagram of WB results. DLBCL, Diffuse large B-cell lymphoma; GSH, glutathione; MDA, malondialdehyde; MMP, mitochondrial membrane potential; ROS, Reactive oxygen species; WB, Western Blotting. Results were expressed as mean ± SE (n = 3). *P < 0.05, **P < 0.01, ***P < 0.001.

Further, HBL-1/DOX cells with CISD2 knockdown using shCISD2 were treated with a gradient of DOX concentrations (0.25 µM–4 µM) for 24 h (Figure 5A). The knockdown of CISD2 significantly reduced the IC50 of HBL-1/DOX cells (1.5685 µM, Fold: 2.5016). When HBL-1/DOX cells transfected with shCISD2 or shCON were treated with 1 µM DOX, shCISD2 treatment significantly reduced cell proliferation (Figure 5B, *P* < 0.05) compared to shCON. After shCISD2 treatment, levels of iron (Figure 5C), MDA (Figure 5E), and ROS (Figures 5F, I) in HBL-1/DOX cells were significantly increased (*P* < 0.05), while GSH levels (Figure 5D) and MMPs (Figures 5G, J) were decreased (*P* < 0.05). In contrast, DOX treatment in shCON-treated HBL-1/DOX cells only slightly increased iron levels (*P* > 0.05, Figure 5C) and decreased GSH levels (*P* > 0.05, Figure 5D), while MDA levels (Figure 5E), and ROS levels (Figures 5F, I) were significantly increased (*P* < 0.05). Compared to shCON, shCISD2-treated HBL-1/DOX cells exhibited decreased protein levels of CISD2, p62, FTH1, and GPX4 (Figures 5H, K, *P* < 0.05) and increased levels of BECN1 and NCOA4 (*P* < 0.05). Furthermore, DOX treatment in shCISD2-treated HBL-1/DOX cells enhanced the downregulation of CISD2, p62, FTH1, and GPX4 proteins, and the upregulation of BECN1 and NCOA4 proteins (*P* < 0.05). These findings demonstrate that inhibiting CISD2 enhances the sensitivity of DLBCL cells to DOX.

### Activation of ferroptosis enhanced CISD2 expression in HBL-1/DOX cells

**FIGURE 6:**
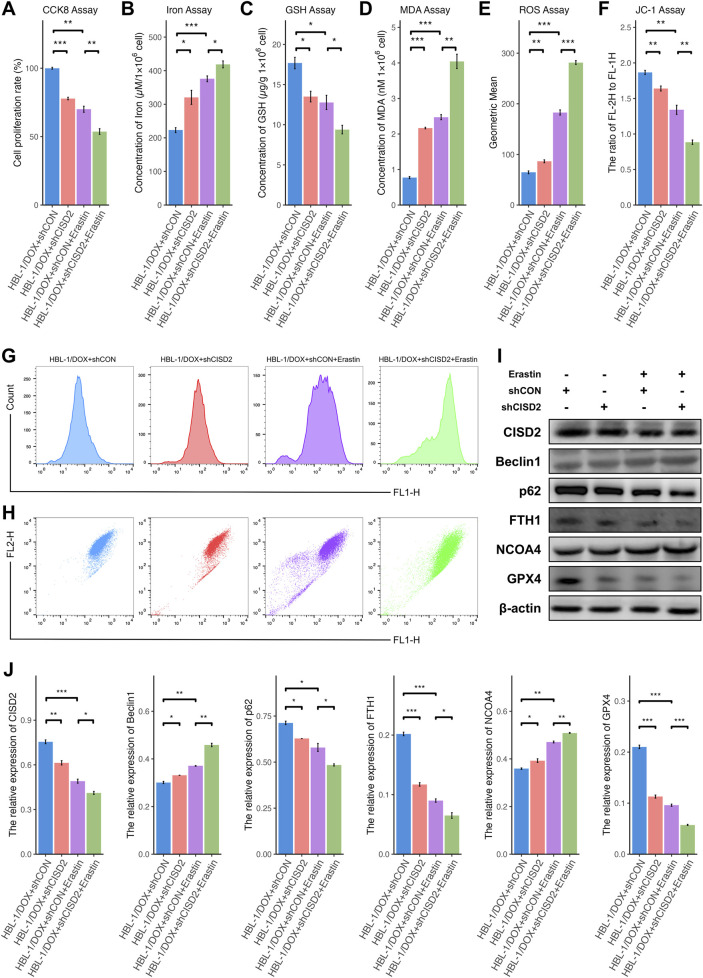
Knockdown of CISD2 enhanced the sensitivity of HBL-1/DOX cells to Erastin. **(A)** The cell proliferation rate after shCISD2 and Erastin treated HBL-1/DOX cells changes. **(B)** Spectrophotometry was used to detect intracellular iron ion levels. **(C)** GSH levels were detected. **(D)** MDA levels were detected. **(E, G)** Intracellular ROS levels in HBL-1/DOX cells treated by shCISD2 and Erastin were detected though flow cytometry. **(F, H)** The MMP levels in HBL-1/DOX cells treated by shCISD2 and Erastin were detected though flow cytometry. **(I)** WB method was used to detect the relative expression of CISD2 in HBL-1 and HBL-1/DOX. **(J)** Schematic diagram of WB results. DLBCL, Diffuse large B-cell lymphoma; GSH, glutathione; MDA, malondialdehyde; MMP, mitochondrial membrane potential; ROS, Reactive oxygen species; WB, Western Blotting. Results were expressed as mean ± SE (n = 3). *P < 0.05, **P < 0.01, ***P < 0.001.

A decrease in cell proliferation was observed in HBL-1/DOX cells transfected with shCISD2 and treated with 10 µM Erastin, compared to the inhibition of shCISD2 in HBL-1/DOX cells (Figure 6A, *P* < 0.05). Additionally, increases in iron (Figure 6B), MDA (Figure 6D), and ROS generation (Figures 6E, G) were induced by Erastin (*P* < 0.05), while decreases in GSH (Figure 6C) and MMPs (Figures 6F, H) were also observed (*P* < 0.05). As shown in Figures 6I, J, treatment of HBL-1/DOX cells with a combination of Erastin and shCISD2 resulted in a decrease in CISD2, p62, FTH1, and GPX4 levels, along with an increase in BECN1 and NCOA4 (*P* < 0.05). These findings suggest that inhibiting CISD2 can enhance the effects of Erastin by promoting increased ferroptosis and ferritinophagy, thereby contributing to the cell death of HBL-1/DOX cells.

## Discussion

Recently, numerous studies have demonstrated that CISD2 is upregulated in various tumors (Zhu et al., 2020; Liao et al., 2021; Yeh et al., 2022), where it promotes tumor cell proliferation by regulating calcium metabolism (Chang et al., 2012b; Shen et al., 2021), oxidative stress, mitochondrial quality control, autophagy, and ferroptosis (Chen et al., 2009; Sohn et al., 2013; Xu et al., 2023). Inhibition of CISD2 has been shown to significantly induce the accumulation of iron and production of ROS in the mitochondria of breast cancer cells, suggesting that CISD2 plays a crucial role in maintaining iron homeostasis and facilitating iron-cluster transfer within mitochondria (Sohn et al., 2013). CISD2 mutations can lead to the loss of [2Fe-2S] cluster function, reducing tumor size and resulting in iron and ROS accumulation in breast cancer cells (Darash-Yahana et al., 2016). Disruption of CISD2 can cause an imbalance in cellular iron homeostasis, increase TXNIP expression, and elevate mitochondrial ROS levels (Karmi et al., 2021). Conversely, overexpression of CISD2 can reduce the accumulation of iron and ROS in tumor cells, enhance the antioxidant system, resist oxidative stress, and promote tumor cell growth (Darash-Yahana et al., 2016; Shen et al., 2021). In this study, DLBCL cell lines transfected with shCISD2 exhibited increased ROS accumulation, decreased antioxidant activity, elevated iron levels, and reduced expression of the ferroptosis-related protein GPX4, indicating that shCISD2 induces ferroptosis in DLBCL cells. Conversely, oeCISD2 transfection produced opposite effects. Our findings suggest that CISD2 may serve as a key ferroptosis-associated gene involved in the regulation of DLBCL development and progression.

Generally, ferroptosis is an iron-dependent form of non-apoptotic cell death (Li et al., 2020; Xie et al., 2023), associated with various diseases, including DLBCL (Zhou et al., 2022). Several studies (Zhang et al., 2023a) have identified ferroptosis-related genes in DLBCL that can predict prognosis and provide targets for precise treatment (Zhou et al., 2022; Wang J. et al., 2023; Wu et al., 2023; Zhang et al., 2023a). Previous research has shown that high expression of CISD2 in DLBCL patients correlates with poor prognosis (Zhang et al., 2023a), suggesting that CISD2 may play a regulatory role in ferroptosis in DLBCL. CISD2, which mainly located in the ER and the outer membrane of mitochondria (Chen et al., 2009; Shen et al., 2021), is involved in mediating the mobilization of iron and [2Fe–2S] clusters between mitochondria and cytoplasm, while regulating mitochondrial iron content and metabolism (Mittler et al., 2019). CISD2 is believed to interact with related genes, participating in the regulation of disease processes (Chang et al., 2012b; Chang et al., 2012a), indicating the complexity of its biological function. Upregulation of CISD2 could reversed ferroptosis induced by Erastin in HepG2 cells (Hou et al., 2023). Overexpression of CISD2 enhances the ability of head and neck cancer (HNC) cells to resist ferroptosis. Conversely, knocking down CISD2 increases lipid ROS and iron levels in HNC cells, thereby potentiating the effects of sulfasalazine-induced ferroptosis (Kim et al., 2018). Actually, several studies have demonstrated that CISD2 is involved in the regulation of drug resistance in tumors (Kim et al., 2018; Li et al., 2021). The combined inhibition of CISD2 and the xCT inhibitor sulfasalazine has been shown to sensitize HNC cells to ferroptosis (Kim et al., 2018). Pioglitazone, a known CISD2 inhibitor, also can induce excessive accumulation of iron and ROS, overcoming ferroptosis resistance in tumor cells (Kim et al., 2018). Silencing CISD2 reduced the cell proliferation of resistant hepatocellular carcinoma (HCC) cells by increasing ferroptosis, while inhibiting both CISD2 and BECN1 decreased ferroptosis in HCC cells (Li et al., 2021). This effect was alleviated by autophagy inhibition (Li et al., 2021). In this study, the induction of ferroptosis using Erastin in DLBCL cell lines was associated with a corresponding decrease in CISD2, accelerated ROS generation, and inhibited GSH levels, highlighting CISD2 as a crucial ferroptotic gene in the occurrence and development of DLBCL. Drug resistance was successfully established in DLBCL cell lines (HBL-1/DOX), with upregulated CISD2 expression observed in HBL-1/DOX compared to HBL-1. Inhibition of CISD2 enhanced the sensitivity of HBL-1/DOX cells to DOX, which was associated with increased ferroptosis and ferritinophagy. Furthermore, HBL-1/DOX cells treated with a combination of shCISD2 and Erastin exhibited greater induction of ferroptosis and ferritinophagy compared to either treatment alone, confirming the synergistic effect of Erastin on CISD2 in promoting ferroptosis in DLBCL cells. These findings highlight the potential of targeting CISD2 to induce ferroptosis and ferritinophagy, thereby increasing cancer cell sensitivity might be a promising strategy for the development of novel anti-cancer therapies.

Specifically, autophagy plays a crucial role in ferroptosis, particularly through the degradation and recycling of ferritin, a process known as ferritinophagy (Gao et al., 2016; Wang H. et al., 2023; Xie et al., 2023). Numerous studies have found that ferritinophagy involves complex interactions with both excessive autophagy and ferroptosis, regulating signaling pathways associated with ferroptosis, including NCOA4-mediated ferritinophagy (Mancias et al., 2015; Wang H. et al., 2023). Increased CISD2 expression has been observed in colorectal cancer (CRC) cells, where it promotes CRC cell proliferation and inhibits both apoptosis and autophagy by activating the Wnt/β-Catenin signaling pathway (Wang et al., 2022). Knockdown of CISD2 significantly accelerated Erastin-induced ferroptosis by increasing Nrf2 ubiquitination and promoting the degradation of the autophagy adaptor p62, while overexpression of CISD2 reduced sensitivity to Erastin (Li et al., 2022). Similarly, Knocking down CISD2 expression significantly increases iron levels in breast cancer cells and promotes iron uptake, leading to autophagy activation (Sohn et al., 2013; Holt et al., 2016). Additionally, CISD2 acts as an ER BCL2-associated autophagy regulator, potentially inhibiting the BECN1 autophagy-initiating complex when BCL2 and CISD2 interact (Chang et al., 2012b). Our results indicate that inhibiting CISD2 may increase BECN1-associated autophagy and induce excessive ferroptosis.

Additionally, while this study demonstrated that CISD2 is involved in the regulation of ferritinophagy and ferroptosis in DLBCL, further research is needed to explore whether CISD2 interacts with ferritinophagy-related proteins such as NCOA4 and FTH1. Moreover, the relationship between the subcellular localization of CISD2 in DLBCL cells and its biological function requires further investigation.

## Conclusion

Finally, this study reveals that elevated CISD2 levels in DLBCL patients may be associated with resistance to the R-CHOP regimen. Inhibiting CISD2 promotes ROS accumulation, lipid peroxidation, mitochondrial damage, and ferroptosis in DLBCL cells, whereas overexpression of CISD2 mitigates ferroptosis. Additionally, increased CISD2 expression was observed in drug-resistant DLBCL cell lines, and knocking down CISD2 enhances ferroptosis, thereby increasing sensitivity to DOX. Mechanistically, the role of CISD2 in DLBCL development and progression may be linked to its regulation of ferroptosis through the induction of ferritinophagy.

## Data Availability

Publicly available datasets were analyzed in this study. This data can be found here: GEO repository, accession number GSE117556.

## Supplementary material

SUPPLEMENTARY FIGURE S1 Observation of DLBCL cell lines transfection A. Schematic diagram of AV plasmid construction; B Using 3 different shRNAs to treat DLBCL cell lines, using snRNA as a control, qRT-PCR method to detect the relative expression of CISD2 (left: SUDHL4, right: HBL1); C oeRNA was used to treat DLBCL cell lines respectively, and onRNA was used as a control, and qRT-PCR was used to detect the relative expression of CISD2 (left: SUDHL4, right: HBL1); D Fluorescence microscope was used to observe the fluorescent staining of AV-transfected DLBCL cells. After transfection of DLBCL cells with shCISD2, shCON, oeCISD2, and oeCON, WB assay detected CISD2 in cells. E Protein expression (left. SUDHL4 cells, right.HBL1 cells), F Schematic diagram of WB detection. AV, Adenoviral; DLBCL, Diffuse large B-cell lymphoma; qRT-PCR, quantitative real-time polymerase chain reaction; WB, Western Boltting. Results were expressed as mean ± SE (n = 3). **P* < 0.05, ***P* < 0.01, ****P* < 0.001, NS. not signifcance.

SUPPLEMENTARY FIGURE S2 Morphology of HBL1 and HBL1/DOX cells were observed under a microscope (left: 10×, right: 40×).
