# Supplementary material for: Inhibition of CISD2 enhances sensitivity to doxorubicin in diffuse large B-cell lymphoma by regulating ferroptosis and ferritinophagy
Source: Front Pharmacol. 2024 Nov 13;15:1482354. doi: 10.3389/fphar.2024.1482354 (PMC11598492; doi:10.3389/fphar.2024.1482354)
Supplement: Supplementary file 1 [file Table1.docx]

Supplementary Table S1 shRNA sequence

| No. | Name | Sequence(5’ to 3’) |
| --- | --- | --- |
| 1 | CISD2-shRNA1-F | GATCCGCTGCGATGGTTCACATAATAATTCAAGAGATTATTATGTGAACCATCGCAGTTTTTG |
|  | CISD2-shRNA1-R | GGATCAAAAACTGCGATGGTTCACATAATAATCTCTTGAATTATTATGTGAACCATCGCAGCG |
| 2 | CISD2-shRNA2-F | GATCCGAGATAATGTGGGTCCACTAATTTCAAGAGAATTAGTGGACCCACATTATCTTTTTTG |
|  | CISD2-shRNA2-R | GGATCAAAAAAGATAATGTGGGTCCACTAATTCTCTTGAAATTAGTGGACCCACATTATCTCG |
| 3 | CISD2-shRNA3-F | GATCCGGATAGCTTGATTAATCTTAATTCAAGAGATTAAGATTAATCAAGCTATCCTTTTTG |
|  | CISD2-shRNA3-R | GGATCAAAAAGGATAGCTTGATTAATCTTAATCTCTTGAATTAAGATTAATCAAGCTATCCG |

Supplementary Table S2 Primer sequences

| Gene | Sequence(5’ - 3’) |
| --- | --- |
| CISD2 | F: GTGGCCCGTATCGTGAAGG |
|  | R: CTAGCGAACCCGGTAATGCTT |
| GAPDH | F: GACAGTCAGCCGCATCTTCT |
|  | R: GCGCCCAATACGACCAAATC |
