# Supplementary figures and images for: Inhibition of CISD2 enhances sensitivity to doxorubicin in diffuse large B-cell lymphoma by regulating ferroptosis and ferritinophagy
Source: Front Pharmacol. 2024 Nov 13;15:1482354. doi: 10.3389/fphar.2024.1482354 (PMC11598492; doi:10.3389/fphar.2024.1482354)

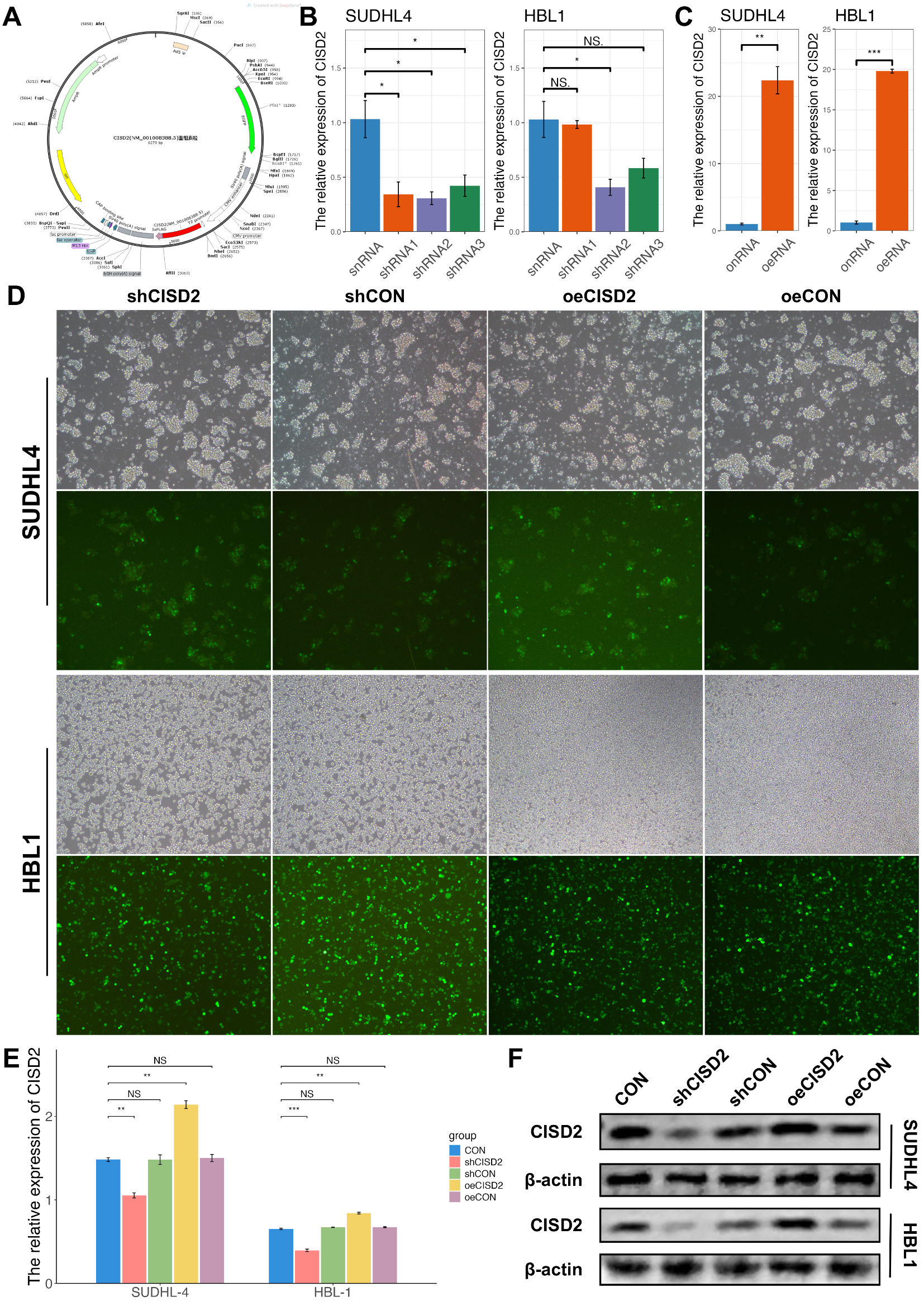

Supplement: Supplementary file 2 [file Image1.JPEG]

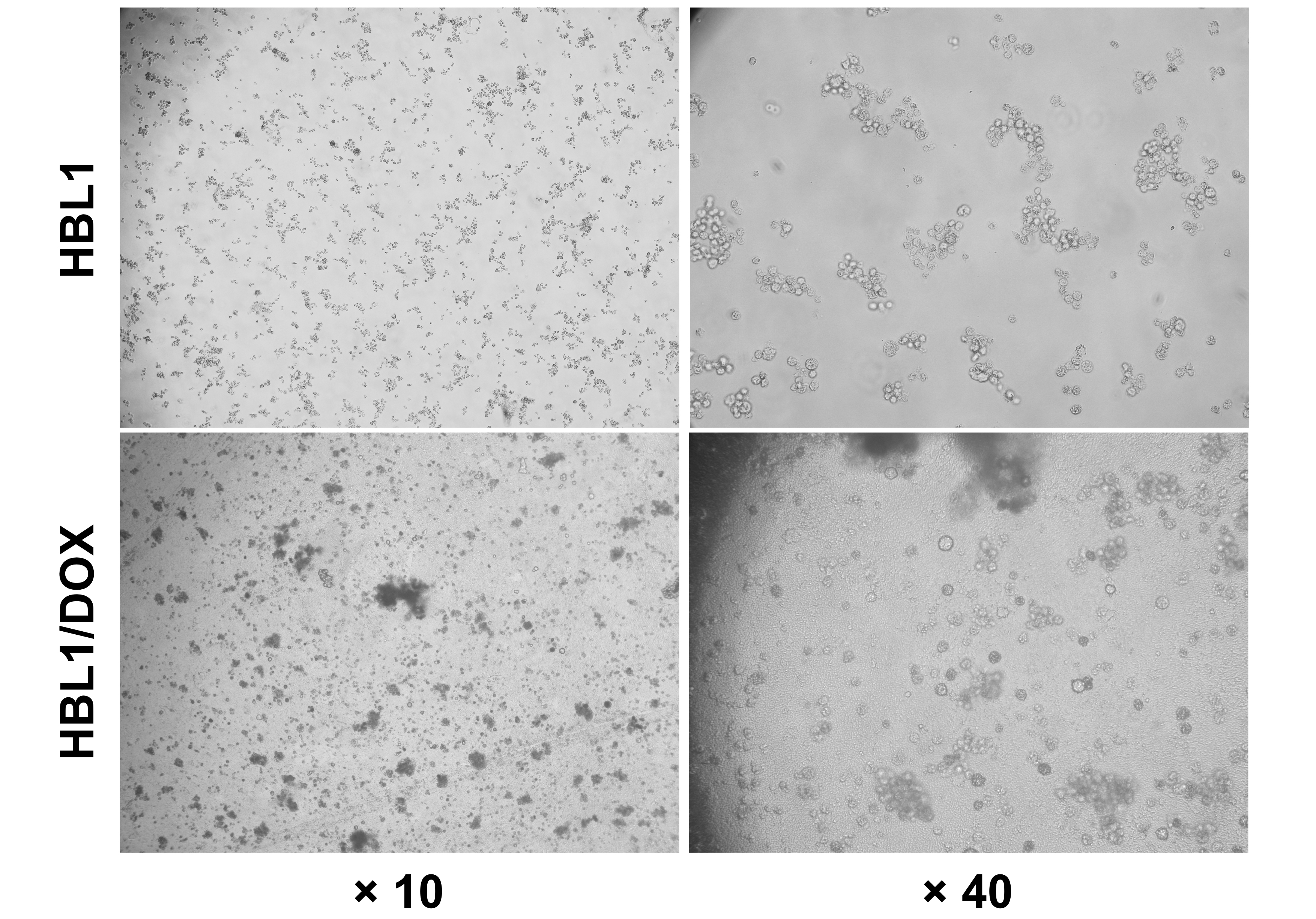

Supplement: Supplementary file 3 [file Image2.JPEG]
